# Quantitative developmental biology *in vitro* using micropatterning

**DOI:** 10.1242/dev.186387

**Published:** 2021-08-03

**Authors:** Guillaume Blin

**Affiliations:** Institute for Regeneration and Repair, Institute for Stem Cell Research, School of Biological Sciences, The University of Edinburgh, 5 Little France Drive, Edinburgh BioQuarter, Edinburgh EH16 4UU, UK

**Keywords:** Microfabrication, Patterning, Self-organisation, Stem cells

## Abstract

Micropatterning encompasses a set of methods aimed at precisely controlling the spatial distribution of molecules onto the surface of materials. Biologists have borrowed the idea and adapted these methods, originally developed for electronics, to impose physical constraints on biological systems with the aim of addressing fundamental questions across biological scales from molecules to multicellular systems. Here, I approach this topic from a developmental biologist's perspective focusing specifically on how and why micropatterning has gained in popularity within the developmental biology community in recent years. Overall, this Primer provides a concise overview of how micropatterns are used to study developmental processes and emphasises how micropatterns are a useful addition to the developmental biologist’s toolbox.

## Abstract (precis)

**Summary:** This Primer introduces micropatterning as a tool to study development and discusses what we have learnt so far with this technique.

## Introduction

Technology development is motivated by the need to overcome specific problems. *In vivo*, the native environment of the cells is complex. Observing cells *in vitro* can be a powerful approach to infer how they might behave *in vivo*. Even so, when grown in a dish, the cells adopt a multitude of shapes; they build colonies of variable forms and densities, and they migrate and often escape the field of observation of the microscope. All this variability can impair quantitative analyses and hide key biological phenomena. More than 50 years ago, S.B. Carter came up with an interesting solution (Carter, 1967); using techniques borrowed from electronics, Carter was able to confine single cells on small adhesive islands (150 µm×100 µm) separated from one another by non-adhesive material. Carter's motivation was quite clear: by controlling cell adhesion, he could achieve a standardised culture and therefore a more manageable complexity that would facilitate the quantification and interpretation of the behaviour of the cells.

**Fig. 1.**
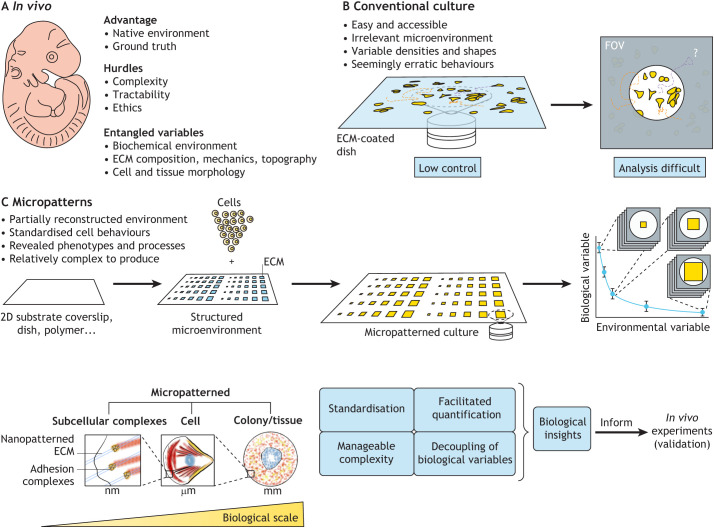
**Micropatterning strengths.** (A) Experiments in embryos can be limited by a number of factors including ethical considerations, low material accessibility and difficulty in disentangling biological variables. (B) Reconstituting developmental processes *in vitro* is an attractive and complementary approach. However, a rationalised control of the *in vitro* microenvironment is required to avoid variable cell behaviours, which confound analysis and may hide important biological processes. (C) Micropatterns offer opportunities to control and uncouple several aspects of the environment including substrate composition, mechanics, geometry and topography. Certain micropatterning techniques can also modulate these variables dynamically. These attributes offer key advantages. For example, careful experimental design with micropatterns enable the precise spatio-temporal perturbation of individual variables, while standardisation facilitates quantitative approaches. Furthermore, as micropatterned cultures are scalable and easy to image, they are compatible with high-throughput applications. Micropatterns range from the nanometre (nm) to the millimetre (mm) scale and thus afford a certain agility to interrogate processes across multiple biological scales. Overall micropatterns can be used to bring biological processes to a manageable yet meaningful level of complexity, offering opportunities to test biological paradigms quantitatively and inform targeted experiments in embryos for validation. ECM, extracellular matrix; FOV, field of view.

The same methodological benefits that Carter introduced with his technique form some of the reasons motivating modern experiments with micropatterns (Box 1; Fig. 1). As fabrication methods have evolved, micropatterns have also emerged in the cell biology literature as a precise tool to both mimic and decouple specific properties of the complex and dynamic cellular microenvironment (Box 2) (Laurent et al., 2017; Ruprecht et al., 2017; Théry, 2010).

**Box 1. Multi parametric quantitative data analysis**

Quantitative experimental design requires control of experimental variables as well as meaningful metrics to monitor the biological process of interest and the effects of eventual perturbations. Here, three examples are provided: (A) front-rear polarity (Théry et al., 2006), (B) fate patterning in embryonic cells (Ostblom et al., 2019; Wisniewski et al., 2019) and (C) collective migration (Jain et al., 2020). In these examples, geometric confinement provides relevant cellular cues and facilitates quantification at the same time. A micropattern chip contains hundreds of cells or colonies that are easy to image. As shapes and sizes are standardised, it is possible to computationally superimpose multiple images together in order to build aggregated data representations showing the average and/or the variation in the signal. This strategy can be repeated for as many stainings, conditions (represented by different colours) or time points (C) as desired to offer a multi-dimensional representation of the biological process. Like all quantitative methods, the technique presents several advantages: it ensures a higher chance of capturing a representative picture of the process (tens of events are aggregated into one representation); quantification gives access to the level of variability in the system, which can provide meaningful information; and the technique may reveal sub-visual information and subtle phenotypes. Geometrical constraints can also facilitate the quantification of phenomena that require near real-time observations, such as the coordination of collective migration (C). Note how the design in this example has made it possible to identify a simple metric (i.e. angular location) to report on a complex phenomenon.

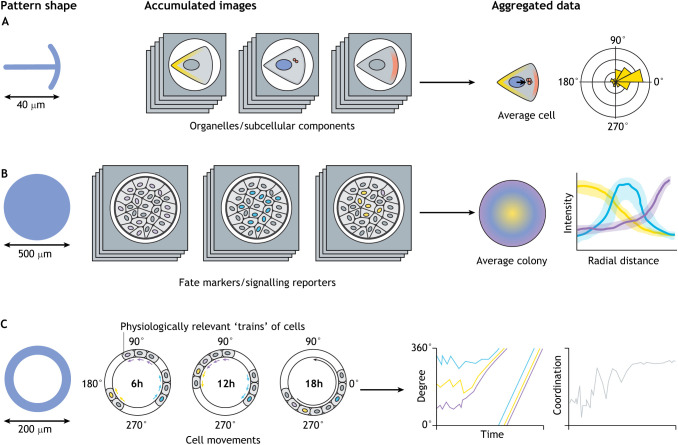

**Box 2. Controlling and uncoupling biological variables with micropatterns**

(A) Micropatterning shines as a tool to dissect the influence of individual extracellular matrix (ECM) properties on cellular processes. For example, various line arrangements can be printed to understand how ECM discontinuities modulate cell shapes and migratory behaviours (Wang et al., 2018). (B) Alternatively, clever single cell patterning designs can be used to dissociate cell shape, adhesion site density and matrix geometry to understand how these variables influence cell fate (Watt et al., 1988), growth (Chen et al., 1997), divisions (Théry et al., 2005) or the intracellular organisation of the cell (Kassianidou et al., 2017; Nardone et al., 2017). (C) As micropatterns can be built on soft substrates, it is possible to decouple matrix chemistry and geometry from matrix stiffness and to measure cell-generated forces using elastic micropost arrays (Lohner et al., 2019). (D) Micropatterns can also mimic *in vivo* spatial constraints. For example, collective migration is influenced by confinement on stripes of various width (Szabó et al., 2016). (E) Micropatterned two-cell systems are useful to decipher morphogenetic processes such as lumen formation (Rodríguez-Fraticelli et al., 2012) and epithelial-to-mesenchymal transition (EMT) (Burute et al., 2017) or to study how the size of cell-cell contacts influence juxtacrine signalling (Shaya et al., 2017). (F) Finally, the geometry and relative spatial distribution of large colonies can be controlled with micropatterns to understand how tissue tension regulates patterns of fate (Ruiz and Chen, 2008) and growth (Nelson et al., 2005), or how autocrine signalling guides branching morphogenesis (Nelson et al., 2006).
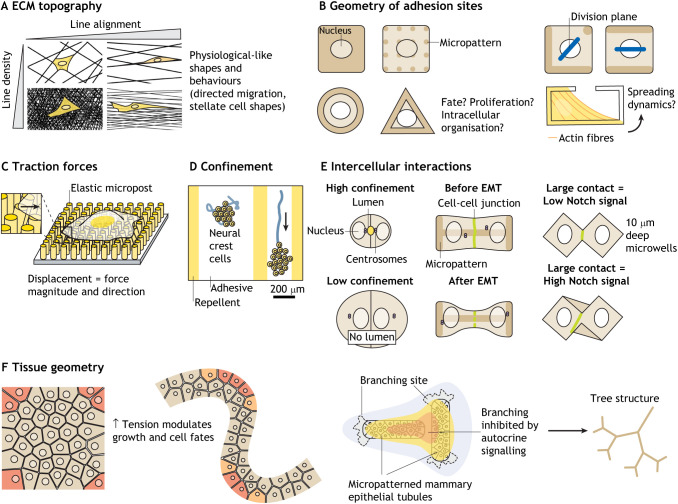

In this Primer, I focus on how micropatterns are used to model early mammalian embryogenesis, not as a replacement for *in vivo* analysis but as a complementary approach that can help to reveal how physicochemical context regulates developmental processes across multiple levels of biological organisation. First, I briefly explain how micropatterns are produced to provide the reader with background for navigating the methods sections of the literature, and then I discuss micropattern models of early embryogenesis and what we have learnt from them so far.

## Fabrication methods

### Photolithography and soft lithography

**Fig. 2.**
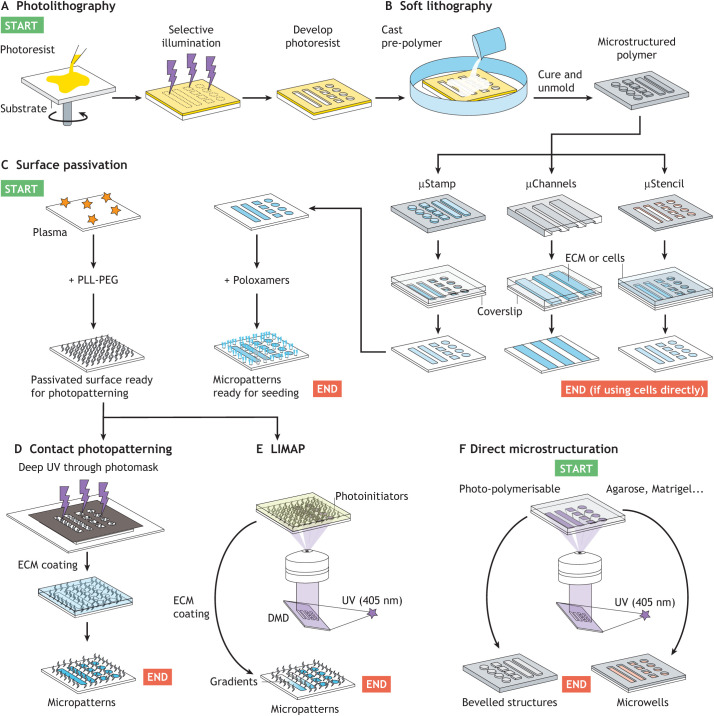
**Micropattern production methods.** (A-F) There exists a broad range of strategies for making micropatterns. Most require a combination of generic procedures indicated in the following panels. The start and end point of different workflows is shown in green and red, respectively. In photolithography (A) the coating of the photoresist is generally performed via spin-coating. Selective illumination can be achieved using a photomask, lasers or a digital micromirror device (DMD). The microstructured photoresist can then be used as a mould for soft lithography (B). PDMS is often used in soft lithography as this polymer self-seals reversibly when placed in contact with another smooth substrate, permitting watertight stencilling and channels to direct the spatial deposition of extracellular matrix (ECM) molecules or cells. If ECM molecules are deposited, surface passivation is needed before cell seeding and can be done using poloxamers (a polymer that adsorbs preferentially on hydrophobic surfaces to form a monolayer of cell repellent molecules) (C, right). Another popular passivation method includes plasma treatment (to activate the surface by ripping-off electrons from the material) followed by adsorption of PLL-PEG (C, left). Selective degradation is then performed with either deep UV through a photomask (D) or using light-induced molecular adsorption patterning (LIMAP) (E), in which ECM density scales linearly with the dose of light allowing for gradient micropatterns. Finally, selective photopolymerisation or photoscission can be performed to create 3D microstructured hydrogels (F). Microstructuration and patterning can be combined to create complex cell environments or microdevices to probe cellular properties (Box 2).

Most micropatterning methods use procedures derived from a technique termed ‘photolithography’. This technique was initially developed to create photographic printing plates as early as 1825, soon becoming popular in the arts. The method was streamlined in the 1950s, when it emerged as a standard method for the microfabrication of various components for the microelectronics industry (Folch, 2012). It is helpful to explain here the principles of photolithography because many micropatterning methods require photolithography as an initial step to build stencils, optical masks and moulds used in the fabrication of micropatterns (Fig. 2).

Photolithography consists of the selective illumination of a thin layer of a photosensitive polymer called a photoresist. Interaction with light chemically modifies the photoresist solubility to a solvent (the developer) enabling the selective dissolution of the illuminated regions (Fig. 2A). This procedure results in a solid microstructure that replicates the light pattern, which can be used for a variety of downstream applications; for example, making photomasks or electronic components. Alternatively, the microstructured photoresist can directly serve as a master mould for casting a curable elastomeric material, such as polydimethylsiloxane (PDMS), to create all sorts of microstructured cell environments or soft devices. Polymer moulding is termed ‘soft lithography’ and forms the initial step of several additive micropatterning methods pioneered by Georges Whiteside and his group in the 1990s (Singhvi et al., 1994). Popular soft lithography techniques include microcontact printing, microfluidic micropatterning or stencilling, which are all aimed at controlling the spatial deposition of cells or biomolecules onto surfaces (Fig. 2B). Soft lithography is a precise, versatile and robust technique that is widely used today, but requires a good amount of expertise and access to a clean room (Folch, 2012).

### Direct photopatterning

More recently, direct photopatterning strategies have been established to make the technique more accessible to non-specialist biology labs. Some techniques use light to control the adsorption of engineered photoreactive proteins (Carrico et al., 2007; Toh et al., 2009). However, due to the protein engineering step, the need for a dedicated photochemistry and also perhaps a lack of awareness in the biology community, these strategies have yet to be widely adopted. Subtractive photopatterning methods, on the other hand, are more commonly found in the biology literature. These techniques consist of first generating a passivated surface, generally via physisorbtion of a polyethylene glycol (PEG)-based polymer, and then using selective illumination to locally degrade the cell-repellent molecules. Extracellular matrix (ECM) molecules can then only adsorb on the irradiated regions of the surface in a subsequent coating step (Fig. 2C-F). For example, contact photopatterning uses deep UV light (<280 nm) through a photomask (Azioune et al., 2010). One advantage of the method is that it can be scaled up to rapidly generate robust micropatterns in parallel in a multi-well plate format for high throughput applications (Tewary et al., 2019). Although custom photomasks can be ordered from several companies, masks are quite expensive to produce and cumbersome to handle.

A powerful alternative is to use an image filter or a digital micromirror device (DMD) docked to a widefield microscope to project a high resolution image (Bélisle et al., 2009; Strale et al., 2016; Waldbaur et al., 2012). This technique, termed ‘light-induced molecular adsorption patterning’ (LIMAP), is made possible thanks to water-soluble and biocompatible photoinitiators that lower the light intensity required to locally degrade the cell-repellent molecules.

### Building complex and dynamic environments

Most of the methods discussed above can be adapted to generate multi-protein patterns (Folch, 2012; Strale et al., 2016; Théry, 2010), as well as ECM density gradients (Ricoult et al., 2015). LIMAP is currently gaining popularity in biology labs because of its simplicity and the fact that companies now sell equipment implementing the technology. LIMAP also makes it possible to generate new adhesive regions while live cells are already attached in culture (Strale et al., 2016). This opens up opportunities to conduct studies on the dynamic response of the cells to a changing environment. Other techniques exist for creating dynamic micropatterns; in fact, the generation of ‘smart’ surfaces and materials, the chemical and mechanical properties of which can be controlled with external stimuli, is an active field of research. For example, biomaterials with a stiffness that is reversibly tunable with light have recently been developed (Liu et al., 2018), as well as many ‘switchable micropatterns’ that respond to light, pH, temperature, electric stimuli or cell-secreted enzymes (reviewed by Badeau and DeForest, 2019; Cimmino et al., 2018; Rapp and DeForest, 2020).

Three-dimensional (3D) micropatterns are also increasingly recognised as a useful tool to understand how dimensionality influences cellular and tissue-scale processes (Fu et al., 2021). Although soft lithography often remains the method of choice for the fabrication of microstructured substrates, direct photo-polymerisation (Yin et al., 2018) and photodegradation (Tsang et al., 2015) of synthetic hydrogels is possible. Recent work has also shown that it is possible to directly ‘carve’ through the polymers and hydrogels commonly used as cell substrates (e.g. agarose, PDMS, Matrigel) using a DMD and a widefield microscope without the need for a dedicated photochemistry (Pasturel et al., 2020). Furthermore, 3D microenvironments can be decorated with biomolecules using biocompatible photolinkers to control substrate topography and surface chemistry independently (Batalov et al., 2021; Pasturel et al., 2020).

In conclusion, making micropatterns still requires some specialist equipment, but the fabrication of micropatterns is slowly becoming democratised thanks to the interdisciplinary efforts of academic labs and their commercial partners who are continuously streamlining the process. Given the variety of micropatterning technologies available, it can be admittedly difficult to identify the best method for a given application. The following reviews include comparative tables that may be helpful to the interested reader: D'Arcangelo and McGuigan (2015), Manzoor et al. (2021), Ruprecht et al. (2017) and Vignaud et al. (2014). Finally, if a high resolution is needed (<100 nm), nanopatterning techniques exist, such as electron beam nanolithography (Changede et al., 2019), as well as nanosphere lithography (Shiu et al., 2018).

## Pluripotent stem cells as a tool to model early mammalian embryogenesis

Pluripotent stem cells (PSCs) are self-renewing cell lines that can differentiate into all somatic lineages *in vitro*. PSCs can be derived from early mammalian embryos (Nichols and Smith, 2011) or from the reprogramming of somatic cells (Takahashi and Yamanaka, 2006). In the past 15 years, PSCs have gained recognition as a powerful experimental system to study complex developmental processes including patterning and morphogenesis. The repertoire of *in vitro* models mimicking aspects of early mammalian embryogenesis is rapidly expanding. Examples include embryo-like stem cell assemblies (Harrison et al., 2017; Rivron et al., 2018; Sozen et al., 2018), neural cysts (Meinhardt et al., 2014; Zheng et al., 2019b), 3D models of gastrulation and axial elongation (Beccari et al., 2018; Brink et al., 2014; Moris et al., 2020; Simunovic et al., 2019; Turner et al., 2017), as well as models of early human embryo implantation (Zheng et al., 2019a). These models offer new, exciting ways to study development through ‘bottom-up’ approaches and are the only experimental systems we can use to study human embryogenesis beyond two weeks of development for ethical reasons (Hyun et al., 2021). Many excellent reviews have recently been published on this topic (Fu et al., 2021; McCauley and Wells, 2017; Shahbazi and Zernicka-Goetz, 2018; Veenvliet and Herrmann, 2020). In the following sections, I discuss those experimental systems that leverage micropatterning and review their advantages and limitations, as well as what we have learned from them so far.

## Micropatterns and the ‘*in vitro* niche’ concept

Using PSCs to study developmental patterning and morphogenesis is a relatively new idea (‘developmental patterning’ refers to the process whereby cells differentiate to form spatially organised domains of cell fates, distinct from the term ‘micropatterning’). Earlier systems for differentiating PSCs, such as embryoid bodies (EBs) or directed differentiation in 2D petri dishes, indicated that PSCs have a propensity for erratic and disorganised differentiation outside the confines of the embryo, even when the cells are provided with a seemingly homogenous and chemically-defined environment. However, in the mid-2000s, a few groups pointed out that the 2D-culture dish is in fact far from being a homogenous environment, because the cells do not spread uniformly in the dish. Evidence from the Zandstra lab showed that variations in local cell densities dictate local concentrations of secreted signals which in turn define the differentiation rate of the cells (Davey and Zandstra, 2006). Thus, micropatterning has been used to calibrate the spatial distribution of the cells in culture. This work has led to the notion that PSCs possess the ability to organise their local environment *in vitro* and that they can respond in a position-dependent manner to their own signals. The term ‘*in vitro* niche’ is sometimes used to describe this self-organised environment in cultures. (Bendall et al., 2007; Lee et al., 2009; Peerani et al., 2007; Peerani et al., 2009). This reference to the *in vivo ‘*stem cell niche’ (haematopoietic niche, for example) illustrates the idea that, within PSC propagation cultures, multiple cell states coexist in a dynamic equilibrium and modulate one another's state and function. Importantly, the *in vitro* niche is defined by a profile of secreted molecules that varies quantitatively between culture conditions (Bauwens et al., 2008; Blin et al., 2018; Kempf et al., 2016) and cell lines (Dziedzicka et al., 2021; Nazareth et al., 2013; Tewary et al., 2019). This has implications for tissue engineering because it provides a causal explanation for interexperimental and interline variability, and also underlines the need for a rational control of initial conditions when aiming to achieve reproducible differentiation outcomes.

A developmental biologist might ask whether the *in vitro* niche corresponds to anything developmentally meaningful. Coincidently, the concept of the *in vitro* niche has emerged concomitantly with the identification of the signalling molecules that drive axis specification in the mammalian embryo (Arnold and Robertson, 2009; Tam and Loebel, 2007). Although it is unclear whether the niche established by PSCs in propagation conditions reflect any developmental process, detailed examination when the cells are cultured in differentiation-permissive conditions in EBs (ten Berge et al., 2008), patterned 2D cultures (Etoc et al., 2016; Morgani et al., 2018; Nazareth et al., 2013; Tewary et al., 2019; Warmflash et al., 2014) unpatterned 2D cultures (Kempf et al., 2016) and 3D gastrulation models (Beccari et al., 2018; Brink et al., 2014; Simunovic et al., 2019; Turner et al., 2017) all indicate that the signalling regulatory networks identified *in vivo* are also functional in equivalent *in vitro* processes. Overall, the ideas discussed above, as well as pioneering 3D organoid work (Eiraku et al., 2011; Sato et al., 2011), have set the stage for the micropatterned-based PSC models of development described below.

## Micropattern models of early mammalian embryogenesis

As PSCs generate their own spatial cues *in vitro*, what might explain the lack of visually apparent spatial patterns in conventional 2D dishes? Possible reasons include the lack of geometrical constraints (we can assume that embryo geometry shapes the distribution of positional signals) and an inadequate chemical environment that may interfere with endogenous self-organisation.

### Gastrulation

**Fig. 3.**
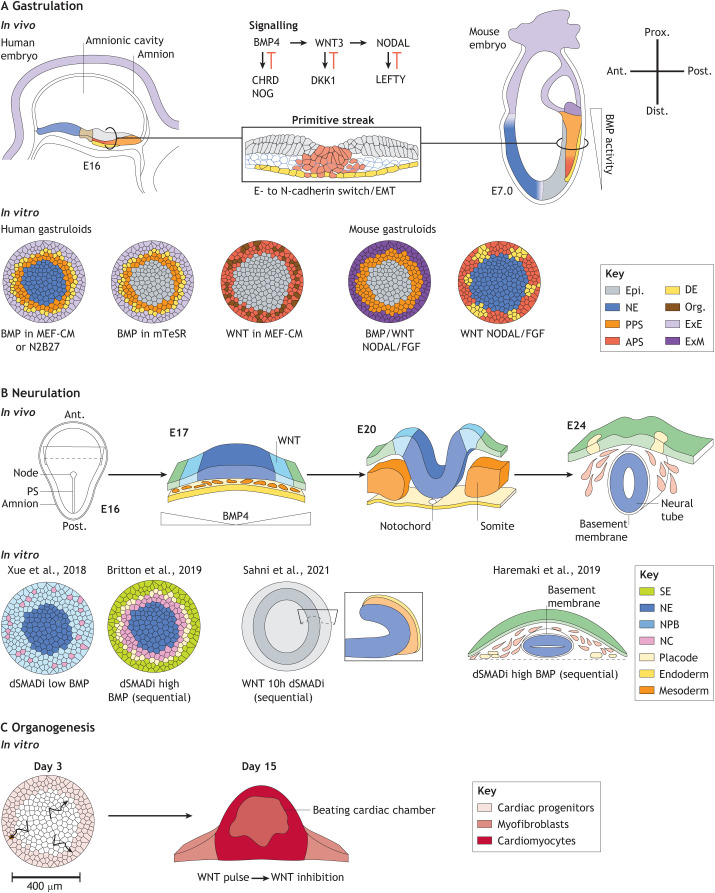
***In vitro* models of mammalian post-implantation development.** (A-C) This figure includes diagrams showing the spatial arrangement of cell fates emerging in micropattern-based models of gastrulation (A), neurulation (B) and organogenesis (C). The location of the corresponding fates in embryos is shown for both gastrulation and neurulation stages. Embryonic day after fertilisation (E) is indicated next to each embryo drawing. (A) An illustration of the signalling network known to regulate patterning during gastrulation *in vivo* and which functions in *in vitro* cultures. In the transverse section of the primitive streak (PS), notice the epithelial-to-mesenchymal transition (EMT) process that accompanies the ingression of the cells in the PS. These characteristics are also found in the PS-like domain of 2D gastruloids. (B) During neurulation, a complex interplay between BMP, WNT and tissue tension is understood to drive the patterning of the ectoderm. These notions have been tested in several models of neurulation. (C) An illustration of the early migratory behaviour (arrows) of cardiac mesoderm before formation of a beating cardiac chamber in a micropattern model of early cardiogenesis. APS, anterior primitive streak; DE, definitive endoderm; Epi., epiblast; ExE, extra-embryonic ectoderm; ExM, extra-embryonic mesoderm; MEF-CM, media conditioned with mouse embryonic fibroblasts; mTeSR, chemically defined medium that includes molecules that activate the NODAL and FGF pathways; N2B27, chemically defined medium; NC: neural crest; NE, neurectoderm; NPB, neural plate border; Org., organiser; PPS, posterior primitive streak; SE, surface ectoderm.

In their seminal article, Warmflash and colleagues remedied both of these issues (Warmflash et al., 2014). They cultured human embryonic stem cells on 1 mm disc micropatterns to provide the cells with geometrical constraints, and used bone morphogenetic protein 4 (BMP4) in conditioned media to act as a differentiation trigger. Within 48 h, BMP4 induced the formation of concentric rings of cell fates with ectoderm in the centre, trophectoderm/amnion-like cells at the periphery (Minn et al., 2020) and mesendodermal fates in between. This system has been referred as a ‘2D gastruloid’ because it recapitulates aspects of gastrulation including signs of primitive streak (PS)-like behaviours within the mesendodermal domain (Fig. 3A). 2D gastruloids have now been reproduced by several other groups (Manfrin et al., 2019; Minn et al., 2020; Tewary et al., 2017) using several different human PSC lines (Tewary et al., 2019) illustrating the robustness of the method. Micropatterned colonies lend themselves well to single cell (sc)RNA-seq studies. Comparison with published datasets from mouse post-implantation embryos and *in vitro* cultured cynomolgus monkey embryos has shown that cell identities in 2D gastruloids reflect early- to mid-gastrula-stage embryos (Minn et al., 2020). Although human 2D gastruloids lack axial mesoderm and extra-embryonic mesoderm, they comprise all the other cell types expected at this developmental stage (Minn et al., 2020).

Several variations of 2D gastruloids have been described that exploit the ability to easily control the chemical environment *in vitro*. For example, a chemically defined medium containing FGF and TGFβ signalling molecules was introduced in Deglincerti et al. (2016), with the resulting difference from the initial protocol that centre cells remain epiblastic instead of becoming ectodermal (Chhabra et al., 2019) (Fig. 3A). Tewary and colleagues have also used N2B27 (another defined medium) and were able to reproduce the patterning observed in the original protocol including the formation of ectoderm at the colony centre (Tewary et al., 2017). Further, a different arrangement of cell fates can be established by directly activating WNT rather than BMP signalling (Martyn et al., 2018, 2019b). This results in the absence of trophectoderm/amnion-like cells and the formation of a PS-like structure directly at the edge of the colony while the centre remains undifferentiated (Fig. 3A). As discussed further below, these platforms have become paradigm experimental systems for investigating the mechanisms underlying germ layer formation in a human context. It should be noted however that 2D gastruloids remain limited in their ability to mimic the complex morphogenetic events that take place *in vivo*. For example, 2D gastruloids do not form the typical tri-laminar structure that gastrulation generates *in vivo* and it will be interesting in future work to investigate what is missing in 2D gastruloids to elicit the collective cell behaviours responsible for morphogenesis.

2D gastruloids have also been adapted to mouse cells in order to enable the direct comparison with mouse embryos (Morgani et al., 2018). It has emerged that mouse cells can be more complex to work with because of the necessity to first direct the cells towards an epiblast-like stage before micropatterning and differentiation. Indeed, mouse cells are thought to be representative of the pluripotent cells of the pre-implantation embryo, whereas human cells are thought to capture a more advanced post-implantation epiblast-like state (Smith, 2017). Therefore, differences in the resulting pattern of cell fates between mouse and human 2D gastruloids may reflect either differences in the initial state of the cells or potential inter-species differences. Interestingly, mouse cells do not generate trophectoderm/amnion cells, whereas human cells do. Nevertheless, mouse 2D gastruloids form spatially-organised domains that include either anterior or posterior PS cell identities depending on whether BMP is included in the cocktail of exogenous signalling molecules. As BMP mutant embryos die at early gastrulation (Mishina et al., 1995; Winnier et al., 1995), it had not been possible to establish the exact contribution of BMP in the PS. These findings thus illustrate how *in vitro* systems can help us circumvent some of the limitations of *in vivo* work and pinpoint potential evolutionary differences.

### Neurulation

Micropattern strategies have also been employed to direct the organisation of anterior tissues (Fig. 3B). Mimicking the anterior epiblast environment via dual inhibition of BMP and NODAL signalling is known to promote PSC differentiation towards anterior ectodermal derivatives (Chambers et al., 2009). On micropatterns, Nodal inhibition and partial BMP inhibition lead to radial patterns of neurectoderm and neural crest tissues (Xue et al., 2018). As BMP is important for patterning ectodermal tissues once the cells are committed to that lineage, the method has been further refined to better simulate these aspects of the *in vivo* environment. SMAD signalling inhibition followed by BMP stimulation sequentially leads to the spatially ordered formation of all major anterior ectodermal tissues including neural ectoderm, neural crest and surface ectoderm, as well as sensory placodes up to a stage equivalent to day 25 of embryonic development (Britton et al., 2019; Haremaki et al., 2019). These systems have been referred as ‘neuruloids’ because they recapitulate the same orderly sequence of events as neurulation *in vivo*. Neuruloids also mimic morphogenetic aspects of neurulation, such as a neural tube-like structure enclosed within a basement membrane that separates it from the migrating neural crest (Haremaki et al., 2019).

The development of neuruloids suggest that early ectodermal morphogenesis is an autonomous process, as non-ectodermal lineages are absent in this system. This contrasts with recent work suggesting that endoderm-derived TGFβ signalling supports the folding of the neural tube in a micropattern model comprising both mesendodermal and neurectodermal tissues (Sahni et al., 2021). I should note that these two observations are not incompatible. In fact, they add to the line of evidence in favour of the notion that development proceeds through the coordination of semi-autonomous developmental units; see Martinez Arias and Lutolf (2018) for a discussion on this topic.

### Organogenesis

Development is associated with rapid 3D growth. As micropatterns impose rigid fixed-sized 2D constraints, there is an *a priori* limit to the growth and therefore developmental stage that can be reached when starting from PSCs. One way to circumvent the problem is to target the differentiation of the cells towards an organ-specific lineage. For example, PSC-derived cardiac mesoderm has been shown to self-organise into beating 3D cardiac chambers on micropatterns (Fig. 3C) (Ma et al., 2015). Other examples include the mechanical patterning of liver (Kaylan et al., 2018) and pancreatic progenitors (Tran et al., 2020), indicating that studying organogenesis on 2D micropatterns is a viable option, as long as a relevant starting population can be identified.

Overall, 2D micropattern models present a range of experimental systems that are complementary to 3D embryoid models. 3D culture does not restrict growth in any of the three spatial dimensions, and is perhaps better suited for modelling multi-tissue organogenesis, whereas 2D micropatterns can be useful to focus on specific sub-processes. One current drawback of 3D models is that very few develop according to a predefined 3D coordinate system; individual cell aggregates often look different from one another and their 3D orientation is neither fixed nor predictable from the start (although tissue engineering techniques might soon alleviate these limitations (Fu et al., 2021; Laurent et al., 2017). On the other hand, micropatterned colonies are standardised, synchronised and easy to image, making them perfectly suited for understanding the mechanisms underlying early patterning events, as I discuss further below. Micropatterned colonies also offer robust assays for development of regenerative therapies, for example to identify the cell lines that are best suited to generate specific clinically-relevant cell types (Nazareth et al., 2013; Tewary et al., 2019). The strengths of micropattern systems also make them excellent platforms for investigating the early developmental origins of certain degenerative diseases (Galgoczi et al., 2021 preprint; Haremaki et al., 2019; Krieger et al., 2019) or to understand chromosomal instability and mosaicism during early development (Yang et al., 2021).

## Mechanisms of pattern formation: diffusible signals

The remarkable robustness of the micropattern systems described above provide opportunities for testing mainstream mechanistic models of developmental patterning in a quantitative manner. Recent studies have notably focussed on how the cells generate spatially organised secreted signals and how these signals in turn regulate fate patterning:

### Self-organised signalling

In all the examples cited above, exogenous signals are added uniformly into the medium, yet the cells respond differentially in a coordinated manner. How does a non-localised signal lead to patterning?

One explanation comes from feedbacks in signalling pathways. To illustrate this point, I mainly focus on 2D gastruloids because these have been the most extensively studied so far. Upon induction, BMP4 stimulates the expression of its own inhibitors Chordin and Noggin (Etoc et al., 2016; Tewary et al., 2017; Warmflash et al., 2014). These secreted inhibitors can diffuse beyond the open borders of the colony while concentration remains high in the colony centre, leading to the formation of a gradient of signalling activity. Culturing the cells in microwells to block the diffusion of inhibitors abolishes patterning (Warmflash et al., 2014). Differential cell responsiveness to BMP4 also contributes in patterning signalling activity. Cells at the periphery are highly responsive to BMP4 as they fail to establish apico-basal polarity and in turn expose BMP receptors towards the medium-facing side of the cell where BMP molecules are free to diffuse (Etoc et al., 2016). Thus, in this system, the geometry of the group coupled with a chemical negative feedback is sufficient to break the initial homogeneity of the system.

**Fig. 4.**
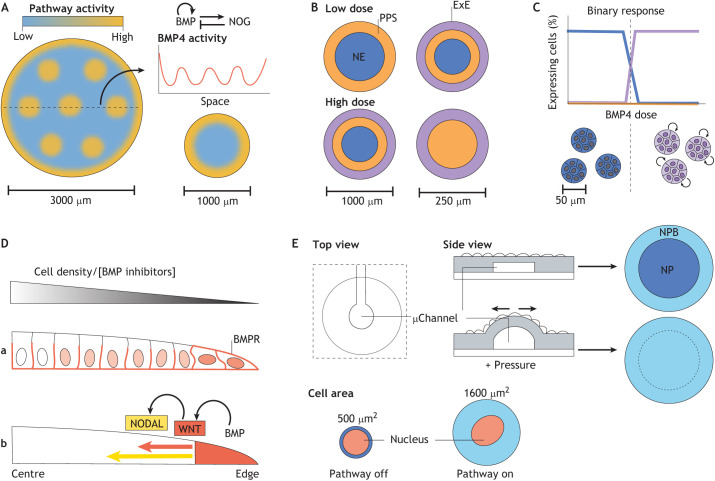
**BMP signalling in micropatterned colonies.** (A) Illustration of the theoretical periodic pattern of BMP pathway activity in large colonies. (B) Illustration of the interdependence between colony size and BMP concentration and its effect on fate patterning. (C) Diagram showing that BMP4 induces only one cell fate in microcolonies (same colours as in B). Small arrows indicate autocrine signalling enforcing the same fates within individual colonies. (D) Side view of a BMP4-treated gastruloid. Cell responsiveness to BMP is highest at the edge of the colony owing to differential receptor localisation (Da). BMP induces secondary signals (WNT, which in turn induces NODAL), which form inward travelling waves of signalling that regulate the emergence of the mesendoderm fates (Db). (E) Description of the experimental approach by Xue et al. (2018) to test the interplay between BMP and tissue tension during neural plate (NP) patterning. Micropatterned colonies were grown on top of inflatable microchannels. Adding air pressure induces tissue folding and increases BMP responsiveness at the colony centre leading to the neural plate border (NPB) fate in the entire colony. Consistently, BMP response depends directly on cell area in single cell micropattern experiments. BMPR, BMP receptor; ExE, extraembryonic ectoderm; NE, neurectoderm; PPS, posterior primitive streak.

One interesting question to ask is whether such a system can scale with different colony sizes. Decreasing colony diameter while maintaining BMP concentration constant leads to the loss of the central domain (Etoc et al., 2016; Warmflash et al., 2014). However, as the system relies on a network composed of an activator and a diffusing inhibitor, one possibility is that the system follows a Turing system model (Turing, 1952) (Fig. 4A). As Turing systems are known to only occur within a precise domain of the parameter space (Maini Philip et al., 2012), Tewary and colleagues have exploited micropatterns to systematically vary signal concentration, differentiation time and colony sizes (Tewary et al., 2017) to explore the hypothesis that BMP and Noggin form a Turing system. Interestingly, interspersed clusters of BMP-responsive cells become apparent in large 3 mm diameter colonies and resemble periodic patterns predicted by Turing equations (Fig. 4A). Furthermore, lowering BMP concentration with colony diameter allows patterning to scale with colony size (Fig. 4B). These results position the Turing model as a plausible mechanism for fate patterning in this system.

### Morphogens revisited

Chemical feedback loops explain how a radial gradient of signalling activity establishes itself despite a uniform signal. However, it does not explain how multiple cell fates result from this gradient. This question brings to mind the French flag problem formulated by L. Wolpert (Sharpe, 2019; Wolpert, 1969). One of Wolpert's solutions to the problem is known as the positional information (PI) model, which posits that the cells can ‘read’ their position within the gradient by adopting distinct cell fates according to discrete signalling intensity thresholds. This model, together with supporting experiments in frogs (Green et al., 1992; Wilson et al., 1997) and fish (Chen and Schier, 2001), has led to the definition of a morphogen as a diffusible molecule that induces multiple cell fates directly without intervention of secondary relay signals.

Micropatterning provides an opportunity to test whether a particular signal meets this definition of a morphogen. Indeed, PSCs can be cultured as individual microcolonies (one to eight cells) in order to limit the accumulation of any potential cell-produced secondary signals (Fig. 4C). Varying the concentration of BMP4 is unable to induce more than one cell fate (trophectoderm/amnion-like) on microcolonies, suggesting that BMP4 does not fulfil the morphogen criteria in this context (Nemashkalo et al., 2017). Rather, the emergence of the mesendodermal lineages in larger colonies requires the combined action of secondary WNT and NODAL signalling (Chhabra et al., 2019; Heemskerk et al., 2019; Nemashkalo et al., 2017; Yoney et al., 2018). Of note, the quantification of intra- and intermicrocolony variation also revealed a community effect (local interactions that sustain BMP signalling) that enforces the commonality of cell fates within individual colonies. This is reminiscent of the Nodal-dependent community effect that coordinates cell ingression in the PS of chick embryos (Voiculescu et al., 2014). It will be interesting, in future work, to examine whether BMP-driven community effects also coordinate differentiation responses in the embryo. This example highlights the power of micropatterns to identify novel mechanisms that are not readily apparent from *in vivo* analysis.

### Signalling dynamics

If patterning is not the result of a classic positional information mechanism, then how do distinct pathways work in concert to orchestrate cell fate decisions in a spatially ordered manner?

Insights into this question have been obtained by taking advantage of the standardisation and synchronicity of patterning in 2D gastruloids. Systematically monitoring the levels of each pathway over time with or without chemical perturbations have shown that signalling is dynamic and follows a precise sequence of events (Chhabra et al., 2019; Heemskerk et al., 2019; Yoney et al., 2018). High BMP4 activity at the periphery induces an endogenous slow inward-propagating wave of WNT signalling (Chhabra et al., 2019; Martyn et al., 2019a), which in turn activates another faster moving wave of NODAL (Heemskerk et al., 2019), propagation of which is moderated by a local feedback loop involving the NODAL inhibitor LEFTY (Liu et al., 2021 preprint) (Fig. 4D). The superimposition of these waves onto fate markers have failed to reveal a direct correspondence between levels of signalling and cell fate (Chhabra et al., 2019). Instead cells may respond to the temporal variations in signal activity, as suggested by several *in vivo* studies revisiting how major signalling molecules transmit robust spatio-temporal information during development (Balaskas et al., 2012; Sako et al., 2016; van Boxtel et al., 2015, 2018). Interestingly, several NODAL response genes are sensitive to the rate of change in concentration of NODAL, whereas others are more sensitive to signal duration (Heemskerk et al., 2019). NODAL response is also dependent on concurrent (Massey et al., 2019) and past WNT activity (Yoney et al., 2018). These findings highlight the necessity to integrate temporal and context dependence of signalling in our understanding of development (Li and Elowitz, 2019), and show that micropatterned colonies are useful to explore the dynamic properties of patterning.

Given the complexity of patterning mechanisms, multi-scale mathematical modelling approaches are particularly useful to determine whether mechanistic models are plausible. Quantitative and multi-parametric data obtained from micropattern experiments have been used to both inform and validate computational simulations. These strategies are already proving fruitful to gain systems understanding of pattern formation (Camacho-Aguilar and Warmflash, 2020; Chhabra et al., 2019; Tewary et al., 2017).

## Geometry- and mechanics-guided patterning

Biochemical cues are not the only extrinsic factors impacting cell fate. Tissue geometry and mechanics play both instructive and permissive roles on patterning. One emerging notion is that the physical state of the cells not only influences the shaping of tissues but also contextualises cell response to biochemical signals to ensure the coordination of morphogenesis with cell fate decisions (Chan et al., 2017).

On micropatterns, cell density, geometry and colony edges impose a pre-pattern in cell polarity and cell tension. This anisotropy contributes to patterning initiation in all *in vitro* models described so far. For example, cell polarity dictates the subcellular localisation of BMP receptors and in turn the competence of the cell to respond to BMP4 (Etoc et al., 2016) (Fig. 4D). Importantly, this mechanism has been shown to operate in the epiblast of mouse embryos as well (Zhang et al., 2019) illustrating how micropattern models can generate hypotheses that can then be tested *in vivo*. BMP pathway activation has also been shown to be conditional on cell tension during ectodermal differentiation, consistent with the idea that tissue folding may regulate BMP responsiveness during neural tube closure (Xue et al., 2018) (Fig. 4E).

**Fig. 5.**
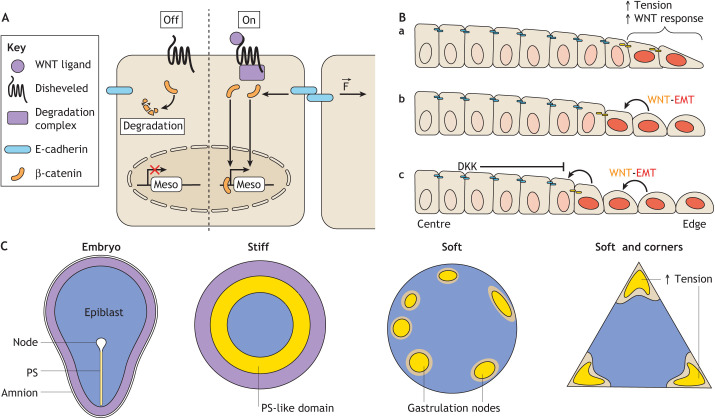
**Convergence of WNT signalling and mechanics.** (A) Diagram illustrating how both canonical WNT signalling and tension at cellular junctions regulate the intracellular level of active β-catenin. Once in the nucleus, β-catenin activates mesodermal genes (Meso). F indicates force applied at the cellular junction. (B) Mechanism of patterning in WNT-treated gastruloids. Although WNT is provided uniformly, cell responsiveness is highest at the periphery, where the cells fail to form a mature epithelium (a). WNT positively regulates its expression at the same time as promoting epithelial-to-mesenchymal transition (EMT) (downregulating E-cadherin) (b). As secreted WNT diffuses, it activates the pathway in neighbouring cells. Concomitantly, EMT in the first cell destabilises junctional β-catenin in neighbouring cells. This phenomenon propagates from cell to cell in an inward direction until a sufficiently high level of secreted WNT inhibitor (DKK) terminates the process (c). (C) Comparative diagram of the patterning in embryos and BMP4-treated human PSC colonies, whether these colonies are micropatterned on stiff plastic dishes or soft hydrogels.

The way physical cues modulate WNT signalling has also been studied in micropatterned colonies (Martyn et al., 2019a; Muncie et al., 2020). Both epithelial integrity and WNT signalling converge onto the regulation of intracellular levels of β-catenin and mesodermal genes activation (Fig. 5A). As WNT promotes its own expression as well as epithelial-to-mesenchymal transition (EMT), the convergence of WNT and forces on β-catenin can lead to fate propagation from cell to cell as revealed by the study of WNT-treated gastruloids (Martyn et al., 2019a) (Fig. 5B). This phenomenon may explain at least partially how the PS maintains itself once initiated. Of note, β-catenin mechanotransduction has been shown in insects (Farge, 2003; Röper et al., 2018), fish (Brunet et al., 2013), cnideria (Pukhlyakova et al., 2018) and human embryonic stem cells (Martyn et al., 2019a; Muncie et al., 2020; Przybyla et al., 2016), suggesting that this pathway is an ancient regulatory mechanism that is evolutionary conserved (Brunet et al., 2013; Pukhlyakova et al., 2018).

In contrast to the embryo, 2D gastruloids remain radially symmetric instead of forming a polarised axis (Fig. 5C). To better understand this phenomenon and gain insights into how forces may regulate PS initiation, Muncie and colleagues have grown micropatterned colonies on compliant hydrogels that mimic the stiffness of the epiblast of avian embryos (Muncie et al., 2020). Interestingly, PS-like domains emerge as discrete ‘nodes’ on micropatterned hydrogels. The location of these nodes correlate with regions of high traction force activity. Consistently, mesoderm differentiation is induced in regions of high tension when the cells are grown on micropatterns of varying geometries (Muncie et al., 2020; Smith et al., 2018). These observations are also in line with microwell experiments showing that contact point with the microwell wall dictates the location of mesoderm induction in EBs (Sagy et al., 2019). It should be noted that varying colony geometry or bringing a group of cells in close proximity with a surface can modulate both the mechanical and the chemical context of the cells. It will be interesting, in future work, to investigate how mechanical cues and secreted gradients are integrated to define patterning in these contexts.

Collectively, these studies suggest that mechanics and geometry may contribute in ensuring the robust location of PS initiation. By dictating epithelial integrity and tissue tension, compliant ECM may prevent spurious initiation, whereas stiff or degraded regions may potentiate PS-promoting signalling activity. This idea is further supported by recent evidence showing that the basement membrane in mouse peri-implantation embryos is remodelled asymmetrically along the anterior-posterior axis (Kyprianou et al., 2020). In addition, experiments with small-scale micropatterns of variable geometries indicate that the local geometry of the PS may fine tune neighbour exchanges at the streak to define which cells eventually ingress (Blin et al., 2018; Burute et al., 2017).

Micropatterns have also proven useful in interrogating the role of scale and geometry during formation of the nervous system. In the embryo, the newly formed neurectoderm folds into a tube (Fig. 4B). When cultured *in vitro*, maturing neural cells form rosettes structures spontaneously both in 2D cultures and in 3D organoids. *In vivo*, there is only one neural tube whereas *in vitro*, the number of rosettes is unconstrained. Micropatterns have been used to identify the optimal geometry and tissue scale that accommodates the emergence of one rosette instead of many (Knight et al., 2018). An interesting observation is that the micropattern diameter at which a single rosette emerges reproducibly differs when PSC are directed towards anterior neurectoderm compared with when they are differentiated towards the posterior neural tube. Although rosette formation is an intrinsic ability of neural tissues, these findings show that tissue-specific geometrical context is needed for development to proceed correctly.

Overall, these studies illustrate how micropatterned models can help us disentangle the respective influence of mechanics, geometry and signalling during complex developmental processes.

## Concluding remarks

These are exciting times to be a developmental biologist. In this Primer, I have illustrated how micropattern systems are enriching an ever-increasing range of *in vitro* models allowing us to revisit fundamental developmental biology questions. Although it is clear that micropattern systems do not fully recapitulate the complexity of embryos, the possibility to ‘isolate’ developmental sub-processes *in vitro* over a range of scales and complexities offers new opportunities to study development in a quantitative manner. Far from reductionists’ ideas, *in vitro* studies remain complementary to *in vivo* analyses and encourage focus on the dynamic nature of biological processes, on the role of the environment in contextualising cell behaviours and on the need for a systems understanding of the relations that explain emergent properties of patterning and morphogenesis.

Future work will likely focus on combining micropattern systems with optogenetics (Krueger et al., 2019), quantitative imaging and synthetic biology (Davies, 2017) to further explore questions surrounding the mechanisms, robustness and plasticity of patterning and morphogenetic processes, perhaps with an evo-devo perspective. As standardisation and automated imaging methods offer the opportunity to acquire rich multi-parametric datasets from micropattern systems, leveraging artificial intelligence may help us infer the rules of development. Furthermore, as continuous efforts are being produced to develop smart and dynamic microenvironments which can evolve in response to cell behaviours (Badeau and DeForest, 2019; Rapp and DeForest, 2020; Uto et al., 2020), we can anticipate that new avenues will emerge to better mimic organogenesis and later development in a standardised manner. Finally, highly standardised *in vitro* models will continue to help us reveal unnoticed, yet important, developmental phenotypes (Galgoczi et al., 2021 preprint; Haremaki et al., 2019; Krieger et al., 2019; Yang et al., 2021) to better understand diseases with a developmental origin, as well as gene-environment interactions during development.
